# Patient-physician relationships, health self-efficacy, and gynecologic cancer screening among women with Lynch syndrome

**DOI:** 10.1186/s13053-019-0123-7

**Published:** 2019-08-13

**Authors:** Kaitlin M. McGarragle, Melyssa Aronson, Kara Semotiuk, Spring Holter, Crystal J. Hare, Sarah E. Ferguson, Zane Cohen, Tae L. Hart

**Affiliations:** 1 Ryerson University, 350 Victoria Street, Toronto, ON M5B 2K3 Canada; 2 Zane Cohen Centre for Digestive Diseases, Sinai Health System, Box 24-60 Murray Street, Toronto, ON M5T 3L9 Canada; 3 Division of Gynecologic Oncology, Princess Margaret Hospital, 610 University Avenue, Toronto, ON M5G 2M9 Canada; 4 Department of Psychiatry, University of Toronto, 250 College Street, Toronto, ON M5T 1R8 Canada

**Keywords:** Lynch syndrome, Gynecological screening, Self-efficacy, Patient-physician relationship, Information

## Abstract

**Background:**

Lynch syndrome, a hereditary cancer syndrome, predisposes women to colorectal, endometrial, and ovarian cancer. Current guidelines recommend that women with Lynch syndrome undergo risk-reducing gynecological surgery to reduce their chances of developing endometrial or ovarian cancer. Little is known about how women with Lynch syndrome perceive gynecological cancer screening, or the psychosocial factors associated with screening attitudes and behaviour.

**Methods:**

This study used a cross-sectional, quantitative design. Using self-report questionnaire data from a sample of women with Lynch syndrome (*N* = 50) who had not undergone risk-reducing surgery, the current study sought to: 1) describe the gynecological cancer screening behaviours of women with Lynch syndrome, as well participant-reported sources of information about Lynch syndrome; 2) examine the extent to which women believe gynecological cancer screening is effective and provides them with reassurance and; 3) assess to what extent relationships with one’s family physician were associated with gynecological cancer screening, perceptions about screening, and health self-efficacy. Data were analyzed using descriptive statistics and Spearman rank-ordered correlations.

**Results:**

Data analyses showed that transvaginal ultrasound was the most common screening behaviour (57%) followed by pelvic ultrasound (47%). Only 22% of participants underwent endometrial biopsy. Patient-physician relationships were related to greater health self-efficacy to manage Lynch syndrome and greater perceived effectiveness of gynecological screening. However, health self-efficacy and better patient-physician relationships were not associated with increased engagement in gynecological cancer screening.

**Conclusions:**

The data suggest that feeling efficacious about managing one’s Lynch syndrome and screening is related to positive interactions and communication with one’s family physician. While this is encouraging, future research should examine educating both family physicians and patients about current guidelines for Lynch syndrome gynecological screening recommendations.

## Introduction

Lynch syndrome (LS), a hereditary cancer syndrome caused by pathogenic variants in the mismatch repair genes including *MLH1*, *MSH2*, *MSH6*, and *PMS2* or by an *EPCAM* gene deletion [1, 2], predisposes women to many types of cancer including colorectal, endometrial, and ovarian cancer [3, 4]. The lifetime risk of endometrial and ovarian cancer in women with LS is estimated at 15–60% and 1–24%, respectively [5]. The National Comprehensive Cancer Network (NCCN) guidelines suggest that women with LS consider surgery, such as prophylactic hysterectomy and/or oophorectomy to lower or eliminate their chances of endometrial or ovarian cancer [5]. Both procedures however, are associated with significant symptom burden including sexual dysfunction, early onset menopause, and body image issues [6–8]. Further, women of childbearing age may not see this as a desirable option.

Among women with LS who have not undergone gynecological risk-reducing surgeries, the NCCN guidelines recommend screening for endometrial cancer with endometrial biopsy every one to two years beginning between ages 30 and 35 [3, 5, 9, 10]. Transvaginal ultrasound (TVUS) for endometrial or ovarian cancer and serum CA-125 testing for ovarian cancer are not considered sufficiently specific or sensitive, although the NCCN guidelines state these screening procedures can be “considered at the clinician’s discretion.” Little is known about how women with LS perceive gynecological cancer screening, or the psychosocial factors associated with uptake and attitudes about screening. In the larger literature on adherence to treatment recommendations among individuals with cancer and other chronic illnesses, high self-efficacy has been associated with greater treatment adherence [11] and higher satisfaction with cancer screening procedures, such as breast mammogram [12]. Therefore, self-efficacy may play an important role in the decision to engage in gynecological screening behaviours for women with LS. Reassurance from screening has been found to positively impact future screening uptake among individuals undergoing colorectal and breast cancer screening [13, 14]. In light of the uncertain value of gynecological cancer screening [10, 15–17], it is yet unknown whether women with LS perceive gynecological screening procedures as effective or reassuring.

In addition to self-efficacy, the relationship between patients and their physicians may also play an important role in engagement in screening behaviours [18]. One study found that compared to colorectal cancer survivors without LS, those with LS were less satisfied with their healthcare providers’ (HCPs) communication, interpersonal treatment, and knowledge [19]. Patients who report a positive working alliance with their physicians (i.e., agree about treatment and trust the physician) view their treatment as more valuable and important [11]. For women with LS, research is lacking as to how relationships with HCPs are associated with uptake and perceptions of gynecologic cancer screening.

The objectives of the present study were to: 1) describe the gynecological cancer screening behaviours of women with LS, as well participant-reported sources of information about LS; 2) examine the extent to which women believe gynecological screening is effective and provides them with reassurance and; 3) assess to what extent relationships with one’s family physician are associated with gynecological cancer screening, perceptions about screening, and health self-efficacy.

## Materials and methods

### Participants

This study was approved by the Research Ethics Boards (REBs) at the Sinai Health System and Ryerson University. Participants were recruited through the Familial Gastrointestinal Cancer Registry (FGICR) at Mount Sinai Hospital in Toronto, ON. Eligibility criteria included: being female, ≥18 years old, a confirmed diagnosis of LS, and prior consent to participate in the FGICR. Women who had previously undergone hysterectomy and oophorectomy were excluded from the current study.

### Procedure

Eligible FGICR participants who consented to be contacted for future research studies were mailed an informational letter, consent form, and questionnaire with a stamped return envelope. All participants provided written informed consent and completed a packet of study questionnaires. Participants received a $20 gift card for their participation.

### Measures

#### Demographic and medical variables

As part of the questionnaire packet, participants self-reported demographic information including age, living arrangement, relationship status, number of children, and employment status, income, level of education, and ethnicity. Participants also provided information about their LS diagnosis date, and the number of relatives diagnosed with LS. Information about type of pathogenic genetic variant and reasons for genetic testing was obtained from the FGICR database.

#### Sources of information about Lynch syndrome

Participants were asked: 1) “To manage your health associated with your Lynch syndrome, where do you get information about screening recommendations?” and 2) “Where do you obtain your general information about Lynch syndrome?”. For both questions, participants selected from the following options: family doctor, internet, surgeon, genetic counsellor, family, gynecologist, newsletter, friends, and gastroenterologist.

#### Gynecological cancer screening behaviours, perceived effectiveness, and reassurance

Gynecological cancer screening behaviours were examined with a 10-item measure created for this study. Participants were asked to indicate ‘Yes’ or ‘No’ as to whether or not they engaged in a given screening behaviour, and were asked to rate the perceived effectiveness of each screening behaviour on a scale from 1 to 5 (1 = not at all effective to 5 = extremely effective). The level of reassurance for specific screening behaviours (i.e., CA 125 blood test, pelvic or TVUS, endometrial biopsy, dilation and curettage, hysterectomy, and oophorectomy) was assessed with a scale created for this study, also ranging from 1 to 5 (1 = not at all reassured to 5 = extremely reassured). This scale had good internal consistency (Cronbach’s alpha = .84).

#### Health self-efficacy

Health self-efficacy regarding cancer risk was assessed with the Communication and Attitudinal Self-Efficacy Scale (CASE) [20]. The CASE is a validated, 12-item scale developed for use with cancer patients. Participants rate each item on a 4-point Likert scale (1 = strongly disagree to 4 = agree). Scores were summed to created three subscales, each with good internal consistency: Understand and Participate in Care (Cronbach’s alpha = .82), Positive Attitude (Cronbach’s alpha = .88), and Seek and Obtain Information (Cronbach’s alpha = .89). Higher scores on each scale indicated higher self-efficacy.

#### Patient-physician relationship

We used an adapted form of the Perceived Involvement in Care Scale (PICS) [21], a 13-item measure assessing patients’ perception of their interactions with their family physician. Each item was adapted to refer to LS instead of a generic medical symptom or treatment. Participants indicated whether they agreed or disagreed with each item. Scores were summed to form three subscales: Patient Information (4 items; Cronbach’s alpha = .87), which measured the degree of information exchanged between physician and patient, Patient Decision-Making (5 items; Cronbach’s alpha = .78), which measured patient involvement in care, and Doctor Facilitation (4 items; Cronbach’s alpha = .59), which assessed physician promotion of patient involvement. Higher scores on the PICS subscales indicated more positive perceptions of interactions with one’s family physician.

#### Beliefs about physician management of Lynch syndrome

Beliefs about patients’ perceptions of their family physician’s effectiveness in understanding and managing their LS were assessed via two items created for this study: 1) “How confident are you that your family doctor understands the cancer risk associated with your Lynch syndrome” (0 = not at all confident to 5 = extremely confident), and 2) “How frequently do you disagree with your family doctor about managing your cancer risk associated with your Lynch syndrome” (0 = never; 4 = always).

### Data analysis

This study used a cross-sectional, quantitative design. Descriptive statistics were used to summarize participant demographic and medical variables, sources of information about LS, and gynecological cancer screening behaviours. Spearman rank-ordered correlations were conducted to determine the association of the patient-physician relationship with the outcomes of gynecological cancer screening behaviours, health self-efficacy, perceived screening effectiveness, and level of reassurance obtained from screening.

## Results

### Baseline characteristics

A total of 54 participants completed questionnaires. Four participants were excluded from analysis because they indicated they had undergone bilateral oophorectomy, hysterectomy or both. Participant demographic and medical factors are listed in Table 1.

**Table 1:** Participant Demographic and Medical Variables

	Mean (SD)/N (%)
Age	39.64 (13.11)
Living Arrangement	
Spouse	36 (72.0)
Self	7 (14.0)
Children	3 (6.0)
Other	4 (8.0)
Relationship Status	
Married or Partnered	37 (75.5)
Separated	2 (4.1)
Widowed	3 (6.1)
Single	7 (14.3)
Children	
Yes	34 (68.0)
Total Number	1.56 (1.40)
Number of Children with LS	.48 (.79)
Employment Status	
Full-Time	30 (60.0)
Part-Time	12 (24.0)
Retired	4 (8.0)
Unemployed	4 (8.0)
Income	
0–40,000	22 (46.8)
40–75,000	11 (23.4)
> 75,000	14 (29.8)
Level of Education	
High School	8 (16.0)
Some College or University	5 (10.0)
College or University Degree	25 (50.0)
Graduate Degree	12 (24.0)
Ethnicity	
White	45 (93.8)
Asian	2 (4.2)
Other	1 (2.1)
Time since LS Diagnosis	3.94 (3.45)
Number of Relatives with LS	4.53 (2.51)
Type of Pathogenic Mutation	
MSH2	19 (38.8)
MLH1	17 (34.7)
MSH6	7 (14.3)
PMS2	3 (6.1)
EPCAM	3 (6.1)

Note: SD (Standard Deviation); N (Total Number of Participants); LS (Lynch syndrome)

The mean age of participants was approximately 40 years old, and most were married or partnered with children. Most participants were employed full-time, highly educated, and white. The average time since participants were diagnosed with LS was approximately four years. Fourteen percent of participants had received genetic testing because of a clinical suspicion of LS (i.e., affected index patients), while 86% received predictive testing for known LS pathogenic variant in the family. MSH2 (38.8%) and MLH1 (34.7%) were the most common type of pathogenic variants in this sample.

### Sources of information about Lynch syndrome

Descriptive statistics summarizing sources of information about LS are displayed in Table 2. Participants most commonly endorsed obtaining general information about LS from genetic counsellors (96%) and the internet (71%). Screening recommendation information was most commonly obtained from genetic counsellors (76%) and family doctors (38%).

**Table 2:** Sources of Information about Lynch Syndrome

	General Information About LS N (%)	Screening Recommendations Information about LS N (%)
Genetic Counsellor	47 (95.9)	38 (76.0)
Internet	35 (71.4)	10 (20.0)
Family	16 (32.7)	10 (20.0)
Newsletter	15 (30.6)	8 (16.0)
Gynecologist	10 (20.4)	15 (30.0)
Family Doctor	9 (18.4)	19 (38.0)
Surgeon	8 (16.3)	10 (20.0)
Gastroenterologist	8 (16.3)	14 (28.6)
Friends	0 (0)	0 (0)

### Gynecological cancer screening behaviours

Descriptive statistics about gynecological cancer screening behaviours are listed in Table 3. The majority of participants reported being watchful of certain symptoms (78%), over half reported obtaining yearly TVUS (57%), and slightly less than half reported obtaining yearly pelvic ultrasound (47%). Approximately 61.2% of participants reported undergoing either TVUS or pelvic ultrasound and 42.9% reported undergoing both TVUS and pelvic ultrasound. Only 22.4% of participants reported yearly endometrial biopsy.

**Table 3:** Descriptive Statistics of Cancer Screening Behaviours and Perceived Effectiveness

Screening Behaviour	Participants Engaging in Screening Behaviour N (%)	Perceived Effectiveness Mean (SD)
I am watchful of certain symptoms	39 (78.0)	3.46 (1.04)
I obtain yearly transvaginal ultrasounds	28 (57.1)	3.41 (1.05)
I obtain yearly pelvic ultrasounds	23 (46.9)	3.64 (1.00)
I get blood tests for the CA125 marker	19 (38.8)	3.00 (1.11)
I obtain yearly endometrial biopsy	11 (22.4)	3.45 (1.13)
I am not aware of any risk reduction steps	2 (4.1)	Not applicable
I had surgery to remove one ovary	2 (4.1)	3.00 (1.41)
I had surgery to remove my uterus	0	–
I had surgery to remove both ovaries	0	–
“D” and “C” - lining of the uterus is removed	0	–

Perceived effectiveness was moderate, with participants rating gynecological cancer screening behaviours that they utilized as “somewhat effective.” On the gynecologic cancer screening reassurance scale, participants reported being “somewhat reassured” to “quite reassured” by screening behaviours (M = 3.58, SD = .68).

### Correlates of screening behaviours

Bivariate statistics (Spearman’s rank-order and chi-square tests) were calculated between health self-efficacy, patient-physician relationships, and engagement in screening behaviours (0 = No, 1 = Yes). No significant correlations were found between health self-efficacy or any  patient-physician relationships scales and participant engagement in gynecologic cancer screening behaviours.

### Correlates of self-efficacy, and perceived screening effectiveness and reassurance

Descriptive statistics for self-efficacy and patient-physician relationship variables are displayed in Table 4. Table 5 displays the Spearman’s rank-order correlations between patient-physician relationships with health self-efficacy, perceived screening effectiveness and screening reassurance. No significant correlations were found between any PICS subscale and health self-efficacy. Significant correlations were found between patient-physician relationships and perceived screening effectiveness. Specifically, endorsing more information exchange with one’s physician, as measured by the PICS Patient Information subscale, was significantly correlated with greater perceived effectiveness of the CA 125 blood test (*r* = .584, *p* = .017), pelvic ultrasound (*r* = .742, *p* = .001), TVUS (*r* = .586, *p* = .003), and endometrial biopsy (*r* = .751, *p* = .012), as well as greater reassurance from screening (*r* = .529, *p* = .024). Being more involved in one’s care, as measured by the PICS Decision-Making subscale, was also significantly correlated with several perceived screening effectiveness items including the CA 125 blood test (*r* = .601, *p* = .018), obtaining pelvic ultrasound (*r* = .625, *p* = .007) and TVUS (*r* = .435, *p* = .049). The PICS Doctor Facilitation subscale was not significantly correlated with any perceived effectiveness screening items.

**Table 4:** Descriptive Statistics for Self-Efficacy and Patient-Physician Relationship

Measure	Mean (SD)	Range
CASE Understand and Participate in Care	3.73 (.44)	1–4
CASE Positive Attitude	3.24 (.78)	1–4
CASE Seek and Obtain information	2.60 (.58)	1–4
PICS Doctor Facilitation	.46 (.31)	0–1
PICS Patient Information	.33 (.40)	0–1
PICS Decision Making	.41 (.38)	0–1
Confident family physician understands the cancer risks with LS?	2.86 (1.17)	1–5
Disagree with family physician about managing your cancer risk?	2.22 (1.17)	1–5

Note: CASE (Communication and Attitudinal Self-Efficacy Scale); PICS (Perceived Involvement in Care Scale); SD (Standard Deviation)

**Table 5:** Spearman’s Rank-Order Correlations between Patient-Physician Relationships with, Self-Efficacy, Perceived Screening Effectiveness, and Screening Reassurance

	CASE Understand & Participate In Care	CASE Positive Attitude	CASE Seek & Obtain Information	Watchful of Certain Symptoms (PE)	PE of CA125 Blood Test (PE)	Undergo Pelvic Ultrasound (PE)	Undergo Transvaginal Ultrasound (PE)	Endometrial Biopsy (PE)	Overall Screening Reassurance
PICS Doctor Facilitation	.273	−.006	.317	−.164	.089	.113	.082	−.071	.134
PICS Patient Information	**.**321	.278	.297	.173	.584*	.742***	**.586** ^**^	.751*	.529*
PICS Decision Making	.248	.134	.331	.122	.601*	.625**	.435*	.392	.215
Confident FP Understands cancer risk	.191	.188	.369**	.179	−.093	.218	.192	.417	.098
Disagree with FP about managing cancer risk	−.169	.051	−.406**	.228	.124	.028	.317	−.028	.281

Note: PICS (Perceived Involvement in Care Scale); FP (Family Physician); CASE (Cancer Self-Efficacy Scale); PE (Perceived Effectiveness). Bolded correlations are statistically significant

*p < .05

**p < .01

***p < .001

Being more confident that the family physician understands one’s LS-related cancer risk was significantly correlated with greater health self-efficacy on the CASE Seek and Obtain Information subscale (*r* = .369, *p* = .010). On the other hand, greater disagreement with one’s family physician about managing one’s LS-related cancer risk was related to lower health self-efficacy on the CASE Seek and Obtain Information subscale (*r* = −.406, *p* = .006). No significant correlations were found between any other patient-physician relationships items, self-efficacy subscales, or perceived screening effectiveness or reassurance.

## Discussion

### Summary of findings

The majority of participants in the present study reported engaging in at least one gynecological cancer screening behaviour. In terms of HCP-recommended screening behaviours, completing yearly TVUS was the most common. Participants reported obtaining LS-related screening information from genetic counsellors and family physicians. In terms of reassurance gained from undergoing TVUS or pelvic ultrasound, the CA-125 blood test, or endometrial biopsy, on average participants reported that they were “somewhat reassured” by these procedures and considered them to be “somewhat effective.” In general, endorsement of better patient-physician relationships was related to greater health self-efficacy to manage LS and to greater perceived effectiveness of various gynecological cancer screening measures, such as the CA-125 blood test, pelvic ultrasound, and TVUS, and endometrial biopsy.

### Sources of information

Genetic counsellors provide crucial informational support to patients with LS. Participants in the present study most commonly reported obtaining general and screening-specific information about LS from genetic counsellors. Genetic counsellors may be the first to inform patients about their LS status, but patients rarely continue to see them in the context of long-term follow-up. Family physicians also play an important role in providing patients with LS with screening-specific information. Over one-third of participants in the present study reported obtaining LS-related screening information from their family physician. Other studies examining sources of information in similar populations have found a much higher prevalence of patients who obtain information from physicians, however, it should be noted that the current study asked about obtaining information from *family* physicians specifically. Prior research shows that most patients with LS who have discussed their condition with an internist or family physician reported that they did not receive gynecologic cancer screening-specific recommendations [22]. In another study, Keinki et al. [23] found that half of the cancer patients in their study who obtained information from a physician were not satisfied with the information received.

### Screening behaviours

Current NCCN guidelines suggest that women with LS undergo endometrial biopsy at least biannually, and that TVUS can also be considered by the healthcare practitioner [3, 5, 9, 10]. The majority of participants in this sample (57%) reported undergoing TVUS however, only 22% reported undergoing endometrial biopsy. These numbers are similar to those reported by Burton-Chase et al. [22], who found that approximately half of their sample of women with LS underwent TVUS and/or endometrial biopsy.

Almost half of the participants in this study (47%) reported undergoing pelvic ultrasound. The most common screening behaviour was being watchful of certain symptoms (78%) and the least common was obtaining endometrial biopsy (22%). However, we did not find any relationship between engagement in gynecological cancer screening behaviours with health self-efficacy or patient-physician relationships.

### Patient-physician relationships, self-efficacy, perceived screening effectiveness and reassurance

Significant correlations were found between better patient-physician relationships and greater health self-efficacy. Specifically, those who felt more confident that their family physician understands the risks associated with LS reported higher self-efficacy to seek and obtain information about LS. We also found participants who disagreed with their family physician about LS management reported less self-efficacy to seek and obtain information about LS. Our findings are consistent with prior studies linking better patient-physician relationships with higher self-efficacy among people diagnosed with cancer [24]. Importantly, both the patient-physician relationship and self-efficacy have been shown to be associated with patient adherence to treatment [11, 25]. Further, among men with prostate cancer who decide to proceed with active surveillance instead of traditional cancer treatment, higher self-efficacy has been associated with lower decision-making conflict [26]. Also in keeping with our findings, difficulty in communicating with health care professionals has been associated with lower self-efficacy about seeking and understanding cancer-related information [27], as well as lower self-efficacy to cope with emotional challenges associated with breast cancer [28].

Significant correlations were also found between patient-physician relationships—specifically greater information exchange and greater patient involvement in care—with the perceived effectiveness of the CA-125 blood test, pelvic ultrasound, and TVUS. Moreover, greater patient involvement in care was associated with more perceived effectiveness of endometrial biopsy as well as with feeling reassured from gynecological cancer screening measures. Prior research shows that poorer patient-physician relationships, such as not trusting one’s family doctor is associated with lower rates of colorectal cancer screening [29], as well as lower intention to obtain breast cancer screening [30]. Men with prostate cancer who have lower confidence in their HCPs also report lower self-efficacy [31]. In addition, a recent review by Peterson et al. [32] reported that the quality of the communication with one’s provider perceived by the patient is associated with greater cancer screening utilization. The current findings suggest that enhancing the exchange of information about LS between patients and providers will be important to improving care for people living with LS.

Some study limitations must be noted. First, this study was limited by its relatively small sample size, and all participants had agreed to partake in a larger familial cancer registry. In general, participants were White, married with children, employed, highly educated, and all had access to health care through the provincial health system in Ontario. Therefore, the findings may be limited in their generalizability to lower income, racialized, and non-insured women. Second, this study examined the constructs of interest using a cross-sectional design and used a number of non-validated measures. Unfortunately, no validated measures existed regarding perceived effectiveness or reassurance of gynecological cancer screening that were appropriate for a LS population. These findings should be considered preliminary, and future studies should consider developing standardized assessments to examine these issues for women with LS. In addition, we asked women to self-report prior gynecological cancer screening, which may under or overestimate actual screening behaviours. Finally, due to limited statistical power, we were only able to examine bivariate correlations using a cross-sectional design, which prohibits us from drawing conclusions about the directionality of significant associations.

## Conclusions

Despite these limitations, our data suggest that feeling efficacious about managing one’s LS and screening is related to positive interactions and communication with one’s family physician. While this is encouraging, it is unclear whether family physicians made the appropriate screening recommendations for gynecologic cancer screening for LS, which would be important to examine in future research. Indeed, it is not uncommon for clinicians to report suboptimal knowledge about LS [33, 34]. A significant proportion of women in our study were not adherent to NCCN screening guidelines. While genetic counsellors are in a critical position to positively influence and inform family physicians about LS-related gynecologic cancer screening, further interventions are needed to help educate these providers and to also improve patient-provider communication among women with LS.

## Data Availability

The datasets used and or analyzed during the current study are not publicly available due to privacy of health information for participants, but are available upon REB approval and data transfer agreement.

## Abbreviations

CASE: Communication and Attitudinal Self-Efficacy Scale
FGICR: Familial Gastrointestinal Cancer Registry
HCPs: Healthcare providers
LS: Lynch syndrome
NCCN: National Comprehensive Cancer Network
PE: Perceived effectiveness
PICS: Perceived Involvement in Care Scale
REBs: Research Ethics Boards
SD: Standard deviation
TVUS: Transvaginal ultrasound
